# The Enigmatic Metallothioneins: A Case of Upward-Looking Research

**DOI:** 10.3390/ijms22115984

**Published:** 2021-06-01

**Authors:** Ahmad Yaman Abdin, Claus Jacob, Lena Kästner

**Affiliations:** 1 Division of Bioorganic Chemistry, School of Pharmacy, Saarland University, D-66123 Saarbruecken, Germany; yaman.abdin@uni-saarland.de (A.Y.A.); c.jacob@mx.uni-saarland.de (C.J.); 2 Univ. Lille, CNRS, Centrale Lille, Univ. Artois, UMR 8181–UCCS–Unité de Catalyse et Chimie du Solide, F-59000 Lille, France; 3 Institute of Philosophy, Saarland University, D-66123 Saarbruecken, Germany

**Keywords:** exaptation, exploratory research, compensation, convergent evolution, metallothionein (MT), moonlighting, multiple realization, protein function, research strategies, scientific discovery, scientific pursuit, upward-looking research, vestiges

## Abstract

In the mid-1950s, Bert Lester Vallee and his colleague Marvin Margoshes discovered a molecule referred to today as metallothionein (MT). Meanwhile, MTs have been shown to be common in many biological organisms. Despite their prevalence, however, it remains unclear to date what exactly MTs do and how they contribute to the biological function of an organism or organ. We investigate why biochemical research has not yet been able to pinpoint the function(s) of MTs. We shall systematically examine both the discovery of and recent research on Dr. Vallee’s beloved family of MT proteins utilizing tools from philosophy of science. Our analysis highlights that Vallee’s initial work exhibited features prototypical of a developing research tradition: it was upward-looking, exploratory, and utilized mere interactions. Since the 1960s, MT research has increasingly become intervention- and hypothesis-based while it remained largely upward-looking in character. Whilst there is no reason to think that upward-looking research cannot successfully yield structure-function mappings, it has not yet been successful in the case of MTs. Thus, we suggest it might be time to change track and consider other research strategies looking into the evolution of MTs. Recent studies in mollusks render research in this direction worthy of pursuit.

## 1. Introduction

After the Second World War, several scientists have been interested in studying the metabolism of metals, especially iron, zinc, and copper in biological organisms. Bert Lester Vallee, who worked at Harvard Medical School and MIT at the time, was a pioneer in the field. After he graduated from New York University with a degree in medicine in 1943, he recognized the potential of spectroscopy to detect metals in biological systems. Regarded as a genius by his colleagues and known for sometimes rigorous questioning of assumptions, Dr. Vallee, as he preferred to be called, had already been reporting extensively about alcohol dehydrogenase (ADH), a metalloenzyme [1]. In the mid-1950s, Vallee came across translations of two publications form Doklady Akademii Nauk SSSR, the official journal of the USSR Academy of Science, dating to 1941 and 1943, and reporting cadmium in several biological systems such as the Aspen tree, algae and other marine species as well as amphibians, reptiles, and even mammals [2]. At first, Dr. Vallee was skeptical about the validity of the data reported. For one thing, cadmium “…never has been demonstrated to be an integral part of a natural product” (1957, p. 4813) [3]. For another, A.O. Vinar (the author of one of those manuscripts) neither mentioned any references in his paper, nor did he employ established methodology. Instead, Vinar reported the results of self-developed colorimetric and spectrographic techniques to measure the amount of cadmium in biological systems. Adding to this potentially problematic methodology, Vallee failed to reproduce some of the experimental results Vinar had reported. For instance, Vallee was unable to induce hypoglycemia in a mammalian species upon injection with cadmium chloride (CdCl_2_) in his own laboratory [2]. Still, Vallee’s interest was sparked. Indeed, he had been studying zinc in biological organisms for years and he could not fail to notice that zinc and cadmium are chemically very similar substances: “The conjoint presence of cadmium and zinc in zinc ores, the difficulties encountered in separating them and ascertaining their identities by chemical means implied that biological matter might discriminate less between their absorption, utilization and perhaps even function than might be thought. Hence, I decided to search for cadmium proteins and enzymes, their biological roles, normal and abnormal metabolism, much as I had proceeded to study the biological role of zinc” (1978, p. 24) [4]. Therefore, Vallee set out to search for cadmium in mammals himself. Together with Marvin Margoshes, he analyzed tissues of human, horse, cow, hog, and sheep kidneys. In 1957, Vallee and Margoshes published a report stating that they actually did identify cadmium in these species [3]. They continued to examine the kidney cortex of equines, which seemed to contain relatively high amounts of cadmium, to try and identify what chemical compound cadmium is part of in the kidney cortex. While the exact relation to zinc remained unclear, Vallee and Margoshes concluded that their results were “…indicative of a low molecular weight protein, probably containing a small number of cadmium atoms per molecule.” (1957, p. 4813) [3] Though its biological function and significance remained completely unclear, this marks the discovery of metallothionein (MT).

It took until 1960 for the term “metallothionein” to be first coined [5]. The name is descriptive in nature. It points to a bond between a metal ion and thionein (a cysteine rich apoprotein) rather than describing a function of these kinds of proteins *per se*, as is the case, for example, with the ADHs discovered a couple of years earlier [6]. Though it was not immediately obvious what MTs can and cannot do, and what purpose they might serve in a living organism, MTs became the focus of an intense and often controversial corpus of research since the 1960s (e.g., [7,8,9,10,11,12]). From a biochemical perspective, there is no question that MTs have unique properties. Besides, they are extremely widespread across taxonomic groups. Based on genetic information, 15 families of MTs have been identified in mammalian and nonmammalian animal species to date and four further families have been identified in plant species [13,14]. Both their biochemical characteristics and their taxonomic prevalence speak to MTs serving some important biological role(s). In fact, a quick search on PubMed Central (pubmed.ncbi.nlm.nih.gov/ accessed 07.04.2021) using just the term “metallothionein” as a query found over 18 thousand hits in the literature category and 34 thousand under the protein category. Six decades after their initial discovery, after literally thousands of studies describing their structure, biochemical characteristics, and tissue distributions, however, the biological function(s) of MTs still remains enigmatic [15,16].

In this paper, we shall discuss both the discovery of and also recent research on MTs from a philosophy of science point of view. We shall systematically analyze the kind of research conducted on MTs so far and which new research strategies might be worthy of pursuit given the *status quo*. Our manuscript is structured as follows. In Section 2 we shall discuss the features that Dr. Vallee’s initial research on MTs exhibits, how these contrast with dominant research strategies in biochemistry and related disciplines such as pharmacology, and how to account for them from a philosophy of science point of view. We shall find that central features of Vallee’s early work are prototypical of a developing research tradition: it was upward-looking, exploratory, and utilized mere interactions. In Section 3, we shall turn to more recent research on the vertebrate MT family. Since the 1960s, MT research has increasingly become intervention- and hypothesis-based while it remained largely upward-looking in character. We shall review three specific hypotheses with respect to which biological functions different MTs might serve. Though hypothesis-based search for one-to-one molecule-function mappings has been successful in other cases, as we shall outline in Section 4, this strategy has not yielded a satisfactory result for MTs to date. Against this backdrop, we suggest, in Section 5, that whilst there is nothing systematically amiss with early or contemporary MT research, it might be time to change track and consider other research strategies to understand the biological and evolutionary role(s) of MTs. Indeed, recent hypotheses proposed in the context of MT research in mollusks support our suggestions. We conclude that studying the evolution of MTs offers a research direction worthy of pursuit for understanding MT function(s) in vertebrates and beyond.

## 2. Classifying Vallee’s Experiments

The dominant picture of research in biochemistry and related disciplines such as pharmacology is that scientists start with a function. They notice, say, that some plants have the ability to convert light into energy and wonder how that works. Or they get interested in how blood pressure is kept constant or muscle growth is being regulated by an organism. In each of these cases, researchers start working from an explanandum (a phenomenon to be explained) to an explanation of how that phenomenon or function is elicited. Paradigmatically, much of the research needed here will be focused on decomposing the system in question, looking at what the different parts of the system do precisely, and how they work together to elicit the explanandum. This analysis may take different forms and run through multiple iterations. Yet, the essential rationale behind any version of this downward-looking approach might be captured by the slogan “divide and conquer!”—researchers are breaking up the system into units, and associating each unit with a specific function [17,18].

### 2.1. Downward-Looking versus Upward-Looking

**Figure 1:**
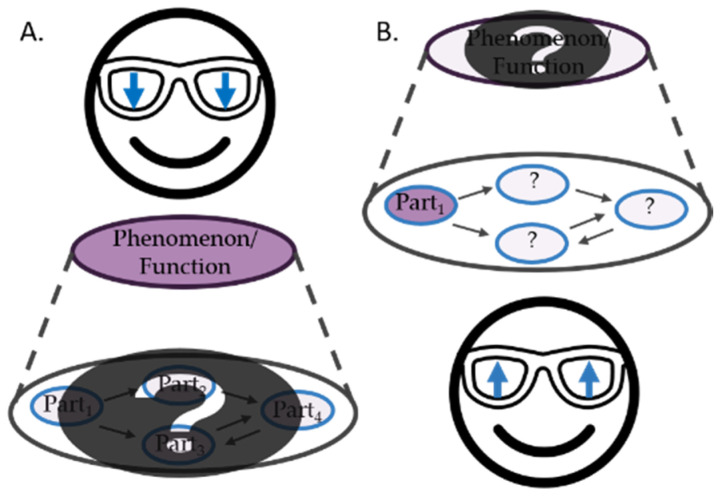
Illustrating the differences between upward looking and downward looking approaches to research. Panel (**A**) represents the downward looking approach, e.g., decomposition. Panel (**B**), represents the upward looking approach, e.g., composition or “re-composition”.

While downward-looking is a good strategy in many cases, this is not all there is to science and scientific inquiry. Recent philosophical accounts of discovery and explanation have been highlighting that identifying the mechanism responsible for a phenomenon to be explained may involve bottom-up or upward-looking research along with downward-looking decomposition strategies (see Figure 1) [19,20,21]. That is to say, rather than just taking a system apart and studying what the different components (parts) do in isolation, researchers are frequently studying individual components while they are looking for what these components do as part of an overall system. If you want to understand, say, hippocampal long-term potentiation (LTP), it is not only interesting to see that *N*-methyl-D-aspartate (NMDA) receptors are part of the mechanism in question. You might want to establish how NMDA receptors contribute to LTP—how they work, how they are triggered, and what happens to LTP if there are no NMDA receptors. Therefore, even if you are starting from an explanandum, LTP in this case, at some point you might need to employ upward-looking research focusing on how a component, in this example the NMDA receptor, contributes to the explanandum.

To illustrate this downward-looking research strategy, we might also consider the case of penicillinase. Almost a decade after Alexander Fleming’s discovery of penicillin in cell cultures of the Gram-positive *Staphylococcus aureus* contaminated by the fungus *Penicillium rubens*, attention was shifted to elucidate antibiotic resistance observed in some bacteria [22]. Researchers prepared purified extracts from crushed penicillin resistant *Escherichia coli*, incubated them with penicillin to then introduce it to bacterial cultures known to be sensitive to penicillin [23]. The processed penicillin demonstrated a significantly lower antimicrobial effect than regular penicillin. When the same kind of extract was prepared from penicillin-sensitive *Staphylococcus aureus* no such effect was observed. Researchers continued to look for the active substance responsible for this effect. In 1940, they identified a protein, termed penicillinase, which they held responsible for inactivating penicillin [24]. Further studies confirming the different sensitivity towards penicillin due to the absence or presence of penicillinase were conducted [25]. Put briefly, we might summarize the case as follows: scientists started searching for what makes bacteria resistant to penicillin by downward-looking research. They crushed resistant bacteria and examined their components. Once they identified a likely candidate, in this case penicillinase, responsible for the phenomenon, here penicillin-resistance, they assessed its relevance in an upward-looking experiment by adding penicillinase to penicillin-sensitive bacteria.

What does this tell us with respect to the case of MTs? With the distinction between upward-looking and downward-looking experiments in place we can clearly recognize Vallee’s early research on MTs, along with most recent MT research we will discuss in Section 3 below, as upward-looking; for it starts from a molecule rather than a function we are trying to explain. This is intriguing. Not because upward-looking research is rare in biochemistry and related disciplines *per se*. But because in the case of MTs it was, and to a large extent still is, the starting point. MTs have not been identified by some sort of downward-looking study. It is noteworthy that not each of the MTS found to date have been identified through upward looking research, though. MT3, for instance, was identified after the observation that extracts from brain cells of Alzheimer’s disease patients supported the survival of rat neuronal cell cultures and was initially called growth inhibitory factor (GIF). Before we continue discussing the nature and prospects of upward-looking research, let us turn to two other distinctions which will be helpful in understanding what sets Vallee’s discovery of MTs apart from much other biochemical research.

### 2.2. Interventions versus Mere Interactions

Experimental work is often characterized in terms of interventions. While the notion is widely used in the empirical sciences to refer to any kind of manipulation, philosophers of science distinguish between different kinds of experimental strategies and manipulations. In this context, the notion “intervention” is reserved for those manipulations which systematically wiggle some, Factor X, in order to elicit changes in another, Factor Y (cf. e.g., see [26,27]). Researchers may intervene, i.e., inhibit or trigger, for instance, into the activity of some part of a system (X) to study how that intervention affects the system’s overall behavior (Y)—or *vice versa* [28]. We may study, say, how penicillinase affects penicillin-resistance or how damaged NMDA receptors interfere with LTP. Intervention-based experimentation is experimentation which manipulates something in order to study the effects it elicits.

Intervention-based research can be contrasted with merely interactive experimentation [29]. Mere interactions are a different form of experimental manipulation; they serve to uncover structures and organization and make accessible features of a system without changing these features. Cases in point are among many others the application of staining techniques, centrifugation of fluids, optogenetics, or—as in Vallee’s case—spectroscopy. In any of these cases, the system is affected, and some aspects of it are being changed, e.g., by staining them. But while interventions are applied to study which effects certain manipulations will have on the system or its parts, mere interactions are employed precisely because they have certain well-known effects, such as attaching marker molecules to certain particles. Unlike interventions, mere interactions do not seek to interfere with what a system or any given part of it does. Rather, mere interactions are sophisticated tools for observation which help scientists uncover features of a system or organism not otherwise accessible.

With this distinction in place, we can recognize, that at least initially, Vallee’s research on the kidneys of various species was a case of mere interactions. He did not seek to change the cadmium content of the tissues he studied to establish what happens when cadmium content is raised or lowered. Rather, he measured it with the technology available to him. He also did not change the kind of protein cadmium was bound to but analyzed its biochemical properties through merely interactive experimentation. Note that while Vallee’s initial mere interactions might be considered a sort of decomposition, they are not downward looking in the sense that he was searching for a component relevant to a specific function he sought to explain (see Section 2.1 and Figure 1).

### 2.3. Hypothesis-Testing versus Exploratory Research

Interventions in the sense just described are frequently carried out to test specific research hypotheses. If we hypothesize that penicillinase plays an important role in penicillin-resistance, for instance, we can test that hypothesis by intervening into, i.e., changing, the presence of penicillinase and observing the effects this has on penicillin-resistance. This approach might be contrasted with exploratory research, viz. research that does not test hypotheses or theories. *Exploratory experiments* are often conducted to map the functional architecture of a system without having a pre-established theory, or to delineate phenomena from their surroundings [30,31,32,33,34]. Exploratory experimentation is very popular before a scientific theory or research tradition is established and before specific hypotheses can be formulated. While it seems tempting to associate exploratory research with mere interactions and contrast it with hypothesis-testing interventions, this dichotomy is premature. In fact, exploratory research can utilize interventions as well. For illustration consider Hubel and Wiesel’s systematic research on cat V1, or Charles Dufay’s “discovery” of the two electricities (positive and negative) [35,36]. In the same way, hypothesis-testing research can utilize mere interactions if, say, we are hypothesizing whether a chemical substance or a biomolecule we can stain for are contained in a sample or not [37].

To summarize the terminology just introduced: experiments can be (i) either exploratory or designed to test specific hypotheses, they can (ii) utilize interventions (studying the effects of a manipulation) or mere interactions (manipulations serving to uncover certain structural features), and they can (iii) be downward-looking, i.e., starting from the phenomenon and searching for what elicits it, or upward-looking, i.e., studying the contribution of a component to a phenomenon or a system’s overall behavior. Without going into the philosophical details here, let us add that classifications with respect to (i)–(iii) are independent of one another. It is worth pointing out, though, that exploratory research and experiments utilizing mere-interactions tend to be more common at the beginning of a research process whereas intervention-based hypothesis-testing research tends to be more common when a research tradition has matured, and results are being published [37]. Against this background, it is little surprising that the dominant view of biochemistry is not only that it focuses on downward-looking research but also that it utilizes interventions and hypothesis testing. Indeed, most research is being conducted and published once a research tradition has matured.

Again, what do we learn from this about MT research? First, we already noted that MT research is mostly an upward-looking search for the function of a biochemical component. And though this may not be the way biochemists typically identify what molecules serve which biological function, this can actually be a successful research strategy, as we shall discuss in Section 4. Second, we can now recognize that Vallee’s research was exploratory in nature—he did not have any pre-established theories and he was not trying to test hypotheses—and that his methodology was based on mere interactions, such as spectroscopy. While these features may also seem intriguing if compared to the large body of published research in biochemistry, it is little surprising to find exploratory and mere interaction-based research where and when a molecule is discovered first. For this simply marks the beginning of a research tradition, in our case the hunt for the elusive function of MTs [16]. The fact that Valle later actually did inject CdCl_2_ into animals to study its effects does not undermine this point; his initial research on MTs was clearly exploratory in nature and based on mere interactions. As we shall discuss in the next Section, MT research has developed into a more hypothesis-testing and intervention-based research tradition since the 1960s. Nonetheless, it remained very much focused on upward-looking research.

## 3. The Enigmatic Role of Vertebrate MTs

Although MTs have been identified in many organisms—many prokaryotes and almost every eukaryote carry genetic information encoding MTs—mammalian MTs are the ones studied most. This is likely because MTs have been identified first in mammalian tissue. Since the initial discovery of MTs, biochemistry has made significant progress with respect to characterizing and classifying vertebrate MTs. By now, we accept that MTs are metalloproteins which have an exceptionally high sulfur content concentrated in 20 cysteine residues and therefore can bind up to seven Zn^2+^ or Cd^2+^ ions or 12 Cu^+^ ions. The high negative charge of the unique metal-sulfur clusters, such as the Zn_4_Cys_11_-α-clusters and Zn_3_Cys_9_-β-clusters in MT-1 and MT-2, is balanced by seven lysine residues, resulting in the equally unique dumbbell shape of the MT proteins which sets them apart from many other biomolecules found in nature [38,39]. Other primary structures of MTs comprising diverse Cys content and coordination, hence varying in metal load and affinities, have been documented [40,41,42].

**Figure 2:**
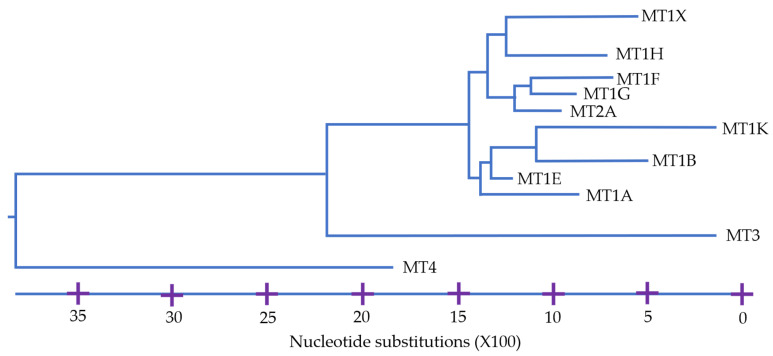
Phylogenetic tree of of protein sequences of MT proteins found in humans. (Adapted from [45] p. 304).

In human cells, three different subfamilies have been identified, i.e., MT1/2 expressed and induced in almost every cell, MT3 in the nervous system and MT4 in the squamous epithelia [43,44]. Phylogenic analyses further categorize these subfamilies into subgroups, i.e., m1P1 and m2P2 as subgroups of MT1 and MT2, respectively, in humans. From these subgroups several human isoforms are specified e.g., MT1A and MT1B. Genetically, human MT1 and MT2 are much less discrete than MT3 and MT4. In fact, MT2 is a member of the MT1 branch, as shown in Figure 2 [13,45]. Though we shall not go into the specifics here, note that similar details have been established already about the biochemical properties of and phylogenetic relations between MTs in other organisms (e.g., [42]). Despite such detailed information, however, the phylogenetic tree of the MT family is still unrooted and researchers’ insights into *the* function of MTs remain very limited to date [15,44,46].

Still, the widespread prevalence of MTs across organisms and their unique biochemical properties suggest that MTs serve, or at least have served, as we shall elaborate below, some important biological role(s). Recent studies have reported a divergence in the function(s) of MTs based on the type of cysteine motifs in invertebrates and vertebrates [40,47]. The new *γ* domain identified in Patellogastropoda, for instance, favors cadmium over zinc and is more resilient against demetallation [40]. Thus, the *γ* domain may be alluding to a specialized metal detoxification function in this natural group. It should also be noted that a role for the noncoordinating amino acids is also being recognized, such as in the case of *Nerita peloronta* MT1 and MT2 (NpeMT1 and NpeMT2) [40,48]. Yet, while the evolutionary forces and developments may be understood better in evolutionarily older and less complex organisms, even here the jury is still out with respect to both the precise function(s) of MTs and their evolutionary history (e.g., [41,49]). We shall return to this in Section 5. For now, we shall focus our attention on thefunction of MT in mammals as this has been the main focus of MT research since their discovery in 1957.

In the late 20th, research has focused on the reactivity and sensitivity of the cysteine ligands towards metalation, oxidation and reduction and the related physiological pathology in mammals [50,51,52,53,54,55]. Three main functions have been proposed over the past 60 years for vertebrate MTs: (i) metal detoxification or toxic metal metabolism, (ii) metal homeostasis or essential metal metabolism, and (iii) protection from oxidative stress (OS). In what follows we shall briefly review these hypotheses and the main research associated to them.

### 3.1. Metal Detoxification or Toxic Metal Metabolism

As the first MTs were isolated from Cd^2+^-poisoned horses, the notion of MTs as detoxifying proteins for heavy metals has been postulated. MT knock out mice are reported to be more sensitive to tissue damage in response to the administration of cadmium, in addition to other in vivo studies reporting an upregulation of MTs after such administration [56,57,58]. A free Cd^2+^ ion inside the cell displaces zinc from MTs leading to an increase in the intracellular concentration of free Zn^2+^. The displaced Zn^2+^ ion is picked up by a zinc sensitive protein, i.e., metal response transcription Factor 1 (MTF-1) which is then translocated to the nucleus of the cell. The induction of thionein, the unmetalated apo form of MT, is then initiated by a transcription promoter called metal response element (MRE). The affinity of MTs to metal ions follows the binding constant of thiolates (Hg^2+^ > Cu^+^ > Cd^2+^ > Zn^2+^). In our example, the excess zinc liberated from MT is then sequestered by this additional thionein. Inside the cell, however, MTs are mostly coordinated to either zinc or copper, two essential and beneficial metals, thus MTs linked to cadmium and other heavy metals are assumed to result from exposure to environmental factors [59].

### 3.2. Metal Homeostasis or Essential Metal Metabolism

Native MTs isolated from mammalian cells are generally bound to zinc and copper ions. Both metals are metabolically important and their intracellular free ion concentrations reside in the picomolar range, despite their micromolar total intracellular content. Zinc has catalytic, structural, and regulative roles in the biology and is a cofactor in about 3000 human proteins [60,61]. As opposed to a previous conception of the metalation mechanism of MTs which was suggested to be of a cooperative nature, i.e., the binding of one zinc ion, for instance, would facilitate the binding of the next zinc ion and so on and there would be a notable absence or instability of partially metalated MTs within the cell, mounting evidence demonstrates that MTs bind metal ions uncooperatively [62]. Such findings have important implications on our understanding on how MTs are involved in the metabolism of essential trace metals. MTs are no longer regarded as zinc storages or thermodynamic sinks, rather as an active component in both the regulative and cellular signaling pathway of zinc, therefore tightly controlling its transient concentration as a protein buffer.

### 3.3. Protection from OS or Antioxidant Scavengers

The antioxidant function of MTs has been proposed due to the exceptionally rich cysteine content (30%) and the fact that OS also leads to upregulation of these proteins [63]. In vitro and in vivo studies, for instance, have demonstrated that ZnMTs scavenge free radicals when treated with hydrogen peroxide (H_2_O_2_) and release Zn^2+^ which in turn leads to the induction of the highly reactive thioneins and MTs through MTF-1 and MRE. Reactive oxygen species (ROS) easily oxidize MTs resulting in their cellular upregulation mediated by an antioxidant response element (ARE) activated by an ARE-binding transcription factor [64]. Increased expression of these proteins mitigates reperfusion injury in the muscle tissues of the heart [65]. It has also been reported that MTs inhibit the copper induced hydroxyl radical HO^●^.

### 3.4. Quo Vadis?

What do we learn from this? Put briefly: despite thousands of studies describing the structure, biochemical characteristics, and tissue distributions of MTs, and even specific suggestions for what role(s) MTs might play in vertebrate organisms, the actual function of the MT family still remains enigmatic [15,16]. 

We might wonder why that is and whether, maybe, there is some sort of systematic problem with MT research. Indeed, some authors have highlighted that MT research faces several non-trivial *experimental* difficulties [7,62]. For instance, applying X-ray diffraction has provided substantial insights into the dumbbell shaped crystalline structure of MTs. Yet such experiments are reported to face significant difficulties and hence, most of the structural characteristics are obtained through NMR analyses which illuminate the coordination of metal-thiolate ligands in the two functional domains but not how they connect and interact [66,67,68]. Another difficulty stems from the fact that d^10^ metals are chromophorically silent in spectroscopic essays. Hence, when studying metal ion transfer scientists substitute zinc with cobalt as a spectroscopic probe [69]. Moreover, the in vitro studies of MTs do not account for the in vivo heterogeneity of these proteins, notably their varying amount and content of metal ions, and the structural models are derived from just one isoform, i.e., Zn_4_Cys_11_-*α*-clusters and Zn_3_Cys_9_-*β*-clusters [7,15]. Due to such unavoidable experimental limitations, one might argue, it is difficult to make general claims about the function of MTs. But is this really a systematic weak point of MT research? Limited, indirect or suboptimal experimental methodologies and tools are common across the empirical sciences. In addition, and quite generally speaking, this does not prohibit progress in principle. Neuroscientists, for instance, study the brain with all kinds of epistemically limited tools. Still, we have a good idea about what certain structures and molecules in the brain do. To be fair, methodological limitations surely are not making it easier to identify MTs’ function(s). Yet that is hardly the sole reason for us not being able to establish what the function(s) of MTs are.

If limited experimental methods are not the issue, one might suspect that identifying MT functions does not currently get off the ground due to the kinds of experiments employed. Based on our exposition in Section 2, however, we can conclude that a focus on exploratory research and mere interactions is neither special nor problematic for an episode of scientific inquiry which lays the foundations of and for a new research tradition. And indeed, recent MT research—as we have just outlined—has moved well beyond this. Over time, there have been theories developed, hypotheses proposed and tested, interventions carried out, etc. So what is left then is the upward-looking character of the overall MT research tradition: if the prototypical approach in biochemistry is to start with a function and search for the molecule(s) relevant for that function, having a molecule and searching for its function might seem a bit like putting the cart before the horse. The attentive reader may suspect this is whence a potential systematic problem might originate. Though, as we shall show in the next section, it is not actually as simple as that either.

## 4. The Case of Ceruloplasmin

To grasp why upward-looking research is not in principle unable to pinpoint a molecule’s function, consider hemoglobin. Hemoglobin is an iron metalloprotein present in the blood cells of almost each of the vertebrates and some invertebrates. It was discovered first in samples of earthworm blood by Friedrich Ludwig Huenefeld (1799–1882) in 1840 [70,71]. While its function was initially unknown, 30 years later French physiologist Claude Bernard (1813–1878) [72] recognized the oxygen carrying property of hemoglobin.

For a more recent example, consider the case of ceruloplasmin. In 1944, Carl G. Holmberg (1906–1991) reported the observation of a unique blue coloration in his insoluble serum fractionation sample, P_I_-fraction [73]. The blue color reminded him of the inactive copper protein isolated by Mann and Keilin in 1938 from the red blood cells of equine serum, named hemocuprein, an enzyme soon referred to as superoxide dismutase (SOD). This inspired Holmberg to identify the copper content of the P_I_-fraction [73]. He quickly realized that this small fraction of the serum contained “…proportionally about five times as much copper as the serum protein as a whole,” ([73] p. 228). Holmberg also noted that the blue color could be oxidized and reduced reversibly, hinting at an enzymatic activity. Today, ceruloplasmin is accepted to play an important role in iron metabolism and as be the main storage for copper in the human body, accounting for 95% of the total body content of this trace metal [74,75].

Both these cases clearly illustrate that searching for a molecule’s functions is, at least sometimes, a successful research strategy in biochemistry. Put slightly differently, that is to say upward-looking research programs are not in principle impossible or systematically flawed. They are viable and can indeed be successful. Thus, the fact that the function of MTs has not yet been identified cannot be blamed on the fact that the search started from the molecule. If this is correct, the reader may wonder, what is left? How come we still cannot say what the function of MTs is? In the next section, we shall offer a few other considerations which might be helpful to advance our understanding of the enigmatic MTs.

## 5. Beyond One-to-One Mappings

From a philosophy of science point of view, searching for “the” one function of a biochemical molecule seems highly idealized. And even if upward-looking research has been successful to associate other molecules with a specific function, that is no guarantee that this strategy will work for the MT family. No doubt, each of the functions suggested for MTs has seemed worthy to pursue at some time (cf. Section 3). Yet, given that there is not much progress being made based on the assumption that MTs serve some sort of unitary evolved biological function, we might have to change track. In what follows, we propose two ways of thinking which might facilitate new research agendas. Note that while our suggestions here originate mainly from considerations of research on a phylogenetically relatively young branch of the MT family, namely vertebrate MTs, recent studies on invertebrate MTs appear to actually support our suggestions [40,44,49].

### 5.1. Multiple Realization, Robustness, and Moonlighting

The first set of considerations we would like to offer revolves around the notion that one-to-one mappings between biochemical components and biological functions might not be realistic. For instance, MTs might serve an important biological function, yet that function might not actually be unique to MTs. What MTs do in biological organisms might also be achieved by other biochemical molecules. For an example of multiple realization, consider the case of hemophilia. Hemophilia is a devastating medical condition characterized by a malfunctioning clotting mechanism, resulting in a potentially life-threatening extended bleeding time. At least three types of hemophilia have been identified, Hemophilia A, B and C, each corresponding to deficiencies in a different clotting factor either genetically or due to pregnancy, autoimmune diseases, or cancer. For a long time, the distinction between the three types went unnoticed [76]. In 1952, Christmas disease, which is now known as Hemophilia B, was suggested [77]. The researchers started from the observations that adding small amounts of normal blood to blood samples from hemophilic patients reduces the clotting time and that in some cases a similar time reduction is observed when mixing two hemophilic samples from different patients. When the isolated antihemophilic globulin, now called as Factor VIII, was added to some of the hemophilic samples no reduction in clotting time was observed. This indicated a variation in the underlying clotting factor responsible for the prolonged clotting period. Further fractionation and precipitation of the blood samples from the Christmas disease patients demonstrated an absence of a specific protein, when compared to normal blood and hemophilic samples lacking Factor VIII which is characteristic of Hemophilia A. This active protein was called thereafter, Christmas Factor or Factor IX.

What is noteworthy about hemophilia is that there is not just one biochemical factor eliciting it, but three different ones. Thus, we see a mapping from one phenomenon (a biological function) to many molecules exhibiting that function (we might consider this a case of *multiple realization*). Now if we assume that this is the case for MTs, too, we would basically have to expect that MTs serve as one of multiple realizers for one or many functions. If so, this might explain why upward-looking research is actually at its limit to uncover the role MTs play in biological organisms; for identifying different biochemical components relevant to a single phenomenon requires knowing what the phenomenon in question is and carrying out downward-looking research. To this end, most of, if not all, the upward-looking MT research done to date will provide useful clues, but it simply will not be conclusive.

Another, though related, reason why it might be so difficult to pin down the biological function(s) of MTs could be that their function(s) might be compensated for easily by other biochemical components or mechanisms if MTs are missing. Or, conversely, MTs might themselves serve a compensatory function in case other important structures or mechanisms fail to operate. The existence of such compensatory mechanisms is at least quite plausible. Ultimately, we know that redundancy and plasticity are important principles contributing to the robustness of phenotypic traits rendering organisms fit for survival [78]. For instance, consider connexins (Cx), the transmembrane proteins which allow the exchange of small molecules between neighboring cells [79]. Cx36 compensates for the function of Cx45 in the retina and Cx32 has been reported to compensate for Cx43 [80,81]. Still, if this way of thinking is correct, the question remains how MT researchers might work their way through the thickets of metabolism in various species to understand the precise role of the MT family.

A third, and the perhaps the most likely option is that although MTs form a seemingly united family of proteins they actually serve a host of different functions in various biological organisms. That is to say, we have a single biochemical molecule that does multiple things in a biological organism. For illustration, consider the case of Glyceraldehyde 3-phosphate dehydrogenase (GAPDH). In glycolysis, GAPDH serves an enzymatic function catalyzing the sixth step of the reaction, i.e., the conversion of glyceraldehyde 3-phosphate to D-glycerate 1,3-bisphosphate. GAPDH has been functionally linked to several non-metabolic functions such as initiation of cell death, transcription of DNA, participation in the antioxidant defense mechanism and metal chaperoning [82,83,84,85]. 

If something like this is also the case for MTs, one might wonder where the multifunctionality of MTs might come from. Recent discussions about protein moonlighting [86,87] suggest an answer: biochemical molecules can actually acquire new and diverse functions through evolution. Proteins can have moonlighting functions based on their localization intracellularly or extracellularly, the types of cells expressing them, the type of substrate available, their quaternary structure (monomers or multimers), or their interaction with other proteins to form more complex structures and multiplicity of binding sites [88]. Within this context, some sort of accidental or systematic biochemical resemblance despite diverse functionality seems at least plausible—and perhaps more feasible than to expect a one-to-one mapping between biological functions and biochemical molecules. If this is correct, searching for “the function” of MTs might not be a productive research strategy. We might be better off asking where the MT family comes from and how it has developed.

### 5.2. Evolutionary Effects: Vestiges, Exaptation, and Convergent Evolution

The second set of considerations we would like to offer originates from the following question: What if the answer to the question of why MTs are so widespread cannot be found in their current but rather in their past biological function? In other words, we would like to point to some evolutionary considerations.

First, we might wonder whether MTs could be vestiges of some sort. That is to say, could they be residues from our evolutionary ancestry which no longer have a biological function? Given how widespread MTs are throughout the animal and plant kingdom, however, MTs would have to go back very far and, there would have to be a common root in the phylogenetic tree of MTs which so far seems to be missing (cf. Section 3). As an example of a vestigial molecular structure in humans consider the cyritestin genes CYRN1 and CYRN2. In other mammals, such as mice, CYRN1 and CYRN2 play an essential role in fertility although they seem to be non-functional in humans [89,90,91]. Other, more conspicuous, vestigial structures in humans are the appendix and the coccyx, i.e. the tailbone.

In light of this, we might consider another option. Namely that the MT family might actually be a result of convergent evolution. That is to say, the same kind of biochemical molecule evolved in different species to perform different, though possibly related, functions. Maybe these functions are somehow linked to metabolizing metals and that is what makes the sulfur-rich metalloproteins such a good candidate molecule for the jobs in question. Contemporary research on invertebrate MTs actually lends support to these evolutionary considerations [40,42,49]. Genetic and biochemical data from mollusks suggests that MTs might have played crucial roles in metal detoxification throughout early evolution. More recently, MTs may have become relevant for homeostasis as living beings adapted to increasingly variable environments [40,92,93,94,95]. Since MTs are somewhat flexible and can change in response to environmental factors, their various forms might have been crucial for organisms to adapt to variable situations of metal bioavailability. Nonetheless, these findings are not yet sufficient to understand the evolution of MTs. Neither is it clear whether and to which extent findings about invertebrate MTs will inform vertebrate MT research. In fact, the evidence reported thus far does not disambiguate between the hypotheses of vertebrate MTs being vestiges of an invertebrate evolutionary history or the result of convergent evolution in response to certain needs for adaptation. A complete phylogenetic tree of the MT family might help to disentangle these scenarios—unfortunately, though, that is not currently available [15,47,96].

Relatedly, MTs might have had some crucial shared biological function which has shifted throughout evolution, i.e., they might be a result of *exaptation*. If so, we nonetheless may find a family of MTs united by a shared biochemistry which now serves different functions in different species or organisms. Calatayud et al.’s suggestion of mollusk MTs having been used for detoxification before they have become important for homeostasis hints in this direction [40]. Indeed, this is perhaps the most promising among our evolutionary considerations: it is effectively a version of the many-functions hypothesis outlined above. Since it is compatible with moonlighting, it comes with a candidate mechanism for how multiple functions have been acquired by the MT family. Further, and as such, it also comes with an evolutionary explanation for the shared biochemistry despite varied functionality within the MT family. Besides, this scenario does not require a common root in the phylogenetic tree because it does not require all MTs to have some sort of common ancestor. At the same time, this scenario is compatible with the idea that most MTs did serve some sort of unitary function at some point in evolutionary history.

Note that the scenarios just described might not be mutually exclusive. In fact, it is possible that MTs first entered the scene independently, then convergently evolved to serve some common or shared function, and then—different organisms faced varied evolutionary pressures as they adapted to different environments—*exapted* to serve different functions across species or organisms. Obviously, this is merely a theoretical scenario for now. Nevertheless, the most recent data on mollusk MTs lends support to the notion that research into the linage-specific and evolutionary old functions of MTs might help us not only to uncover the ancient functions of MTs but also their prevalence and function across animal phyla today, including evolutionarily younger and more complex organisms such as vertebrates. Thus, modifying the research strategy for investigating the function(s) of MTs by focusing on their evolutionary history provides a very promising outlook.

## 6. Conclusions

Research on MTs has started initially from the recognition that these proteins are extremely prevalent across the animal and plant kingdoms. “Why do so many species have MTs?” was thus interpreted as a question about MTs’ function, demanding researchers to find out what MTs are good for. Nonetheless, having an important biological function today is not the only possible reason for why some biochemical molecule may be abundant across most of the taxonomic kingdoms. The hypotheses we outlined in Section 5 acknowledge that and we suggest that—rather than searching for the function of MTs—we should ponder about possible origins of MTs to explain their prevalence. Focusing on the evolutionary history of MTs rather than their contemporary function has the potential to resolve the question of why MTs are so prevalent across species. As such, it has a high problem-solving capacity or programmatic character [97] and thus presents a pursuit-worthy research strategy likely to elicit scientific progress (e.g., [98,99]).

Of course, none of the suggestions we put forward in the previous section are full-fledged theories of vertebrate MT function. Yet, they might offer useful hypotheses for experts in the field to pursue—especially given that similar considerations have already been offered in the context of evolutionary studies of invertebrate MTs. What we can conclude at this point is the following: Despite the special circumstances of the discovery of MTs, there is probably nothing systematically amiss in the research tradition. What appears so intriguing about Vallee’s early work is actually characteristic of discovery episodes leading towards the foundation of a research tradition: upward-looking and exploratory research involving mere interactions. Besides, the upward-looking search for functions is—at least in principle—a viable strategy which has been successful in some cases, as discussed in see Section 4. It is neither in principle fruitless nor systematically flawed.

Nonetheless, it looks as if MT research has not been going forward as much as biochemists would have liked it to and the biological role(s) of MTs still remain enigmatic, especially for vertebrates. This should motivate us to seek a different direction for contemporary MT research. The suggestions we presented in Section 5 provide some options. That they converge with recent empirical work on the evolution of mollusk MTs highlights two things: first, it shows that a systematic philosophical analysis of empirical research may offer support for empirical research decisions. Second, it emphasizes the pursuit-worthiness of evolutionary MT research. That is to say, it renders evolutionary approaches even more likely to solve the research problem at hand and to elucidate why MTs are so prevalent, what precisely they do, and where they come from—in ancient and also in recent animal phyla.

## Data Availability

No new data were created or analyzed in this study. Data sharing is not applicable to this article.
